# The Intraoperative Fabrication of PMMA Patient-Specific Enophthalmos Wedges and Onlays for Post-Traumatic OZC Reconstruction

**DOI:** 10.3390/cmtr18020029

**Published:** 2025-05-29

**Authors:** Layton Vosloo

**Affiliations:** Independent Researcher, Johannesburg 2000, South Africa; vosloolayton@gmail.com

**Keywords:** post-traumatic orbital defect, orbital reconstruction, enophthalmos, patient-matched implant, 3D printing, polymethylmethacrylate

## Abstract

Objective: Trauma is a leading cause of enophthalmos, typically resulting from an increase in the volume of the bony orbit. The general consensus is that post-traumatic primary deformity repair should aim to restore the premorbid volume, shape, and cosmesis of the orbitozygomatic complex (OZC). This study aims to utilise novel three-dimensional (3D) printed patient-specific moulds to intraoperatively fabricate enophthalmos wedges and onlays using polymethylmethacrylate (PMMA) bone cement to reconstruct the OZC. Methods: A total of seven patients underwent digital surgical planning using Freeform software to virtually correct orbitozygomatic complex deformities guided by a design algorithm. Three-dimensionally printed nylon patient-specific moulds were used intraoperatively to fabricate enophthalmos wedges and/or onlays using an industry-standard PMMA bone cement. Clinical examination and application of the proposed design algorithm determined that enophthalmos wedges were indicated for four patients, with one also requiring an onlay; and periorbital onlays were required for the three remaining patients. Results: Hertel exophthalmometry at a mean follow-up of 19.1 months demonstrated good outcomes in the correction of post-traumatic enophthalmos and hypoglobus and with patients reporting good subjective cosmetic results. Patients 5 and 7 had follow-up three-dimensional computed tomography (3D-CT) to confirm correct placement. Conclusion: The use of patient-specific PMMA wedges and onlays, fabricated intraoperatively with the aid of 3D-printed moulds, offers a reliable and effective approach for correcting post-traumatic enophthalmos and hypoglobus. This method allows for the restoration of orbital volume and anatomical contours, addressing both functional and aesthetic concerns. Our results demonstrate that this technique yields favourable outcomes.

## 1. Introduction

Enophthalmos, defined as the posterior displacement of the ocular globe within the bony orbit, most commonly results from inadequately treated orbital fractures, and it is frequently accompanied by hypoglobus and diplopia as a result of disruption to the orbital floor. The principal mechanism underlying post-traumatic enophthalmos is the displacement of a relatively constant volume of orbital soft tissue into an enlarged bony orbit, leading to an orbital volume mismatch [1].

Unlike other orbital pathologies, post-traumatic enophthalmos arises primarily from structural alterations in the bony configuration of the orbit, rather than significant changes in the orbital soft tissue volume [2,3,4]. Consequently, an effective treatment is primarily to restore the premorbid orbital volume and globe position and secondarily, to address soft tissue deficiencies [5]. Traditional autologous and alloplastic implants often fail to fully restore orbital volume, leading to persistent functional and aesthetic deficits.

Accurate reconstruction requires meticulous clinical assessment, adequate imaging, and precise measurement of the orbital defect. To this end, a proposed implant design algorithm provides a systematic approach to delineate the borders of the bony defect, quantify the extent of injury, and facilitate the intraoperative fabrication of patient-specific implants. The algorithm further incorporates a secondary phase, introducing an intraorbital volume overcorrection factor (OF) to address interorbital soft tissue mismatches as a result of orbital lipoatrophy. This mismatch may result from (1) a primary postoperative infection involving adjacent structures, such as a defect in the medial wall in the presence of sinusitis; (2) from delayed repair of an orbital floor blowout fracture with a ruptured periorbita and herniation of the contents into the maxillary sinus; or (3) inadequately reduced OZC fractures.

**Figure 1:**
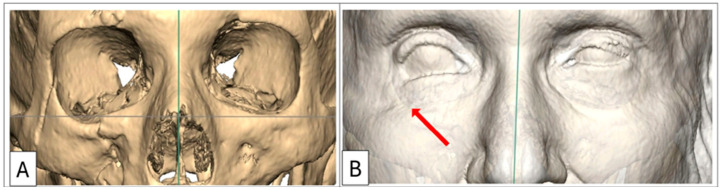
Case 1: Preoperative 3D-CT scan demonstrating lower eyelid retraction due to traumatic deformity (red arrow), requiring an infraorbital rim onlay implant and a lateral tarsal strip canthoplasty. Inferior displacement of the right lateral and infraorbital rim, (**B**) bone window, (**A**) soft-tissue window.

A comprehensive assessment of the orbitozygomatic complex allows for the design and inclusion of adjunctive implants, such as orbital rim and malar onlays, into the reconstruction process. Orbital rim and malar implants primarily address cosmesis. Therefore, orbitozygomatic reconstruction not only addresses functional aspects but also addresses cosmetic concerns such as upper eyelid pseudoptosis as a consequence of globe recession, which may alter the upper eyelid contour and deepen the superior tarsal fold [6]. Similarly, lower eyelid malposition—manifesting as retraction—often arising from a combination of scarring, soft tissue contraction, and, critically as in Case 1, due to inferior displacement of the lateral and infraorbital rim, can also be addressed (Figure 1) [7,8].

This multi-phased implant design algorithm ensures the restoration of orbital volume, the correction of the globe’s position, and the mitigation of cosmetic concerns.

## 2. Materials and Methods

This single-centre study included seven patients who underwent secondary orbitozygomatic complex reconstruction at a level 1 trauma hospital in Johannesburg, South Africa. All procedures were performed by one of two attending maxillofacial surgeons, with the author participating as either first or second surgical assistant and holding the delegated role of intraoperative implant fabrication. The author, a salaried employee of the mould manufacturer (Selective Surgical), functioned as an independent researcher in this context.

**Figure 2:**
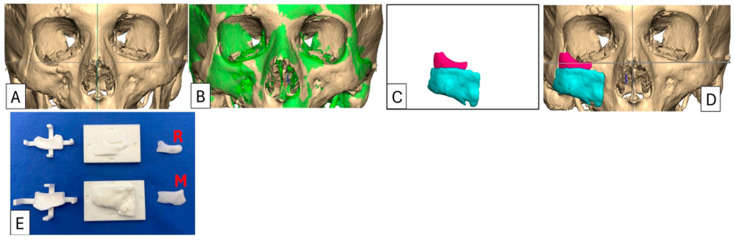
Case 1: Preoperative 3D-CT scan and planning, intraoperative moulds and implants. (**A**) As-scanned anatomy. (**B**) Left orbitozygomatic complex digitally mirrored and superimposed (green) over right orbitozygomatic complex. (**C**) The implant is determined by subtracting the relevant as-scanned anatomy from the unaffected mirrored anatomy i.e., (**B**) − (**A**) = (**C**), resulting in the proposed 2-piece implant design. (**D**) Implant design positioned onto the as-scanned anatomy. (**E**) The negative moulds represent the volumetric deficiency designed from the form of (**C**) and which is populated with PMMA cement. Orbital rim implant (R) and malar onlay (M). For this case, the implant was designed in two sections to facilitate insertion. The orbital rim implant was inserted through a transcutaneous infraorbital incision, and the malar onlay was inserted through an intraoral approach. Both implants were secured with titanium 1.5 mm diameter screws.

This patient group consisted of seven individuals (five males, two females) with a mean age of 35 years (range 25–65), all of whom presented with unilateral post-traumatic OZC deformities. Indications for surgery included enophthalmos, asymmetry, and persistent diplopia. All patients underwent secondary reconstruction using the described workflow, which represents the iterative development of the design algorithm. To ensure the accurate measurement of postoperative improvement, only patients with unilateral blowout fractures and/or unilateral displacement of orbitozygomatic structures were included (Table 1). The normal contralateral anatomical morphology was digitally mirrored and superimposed over the traumatised orbit, enabling precise determination of the extent of displacement (Figure 2). This allowed the contralateral side to be used as a reference for planning during the initial development of the algorithm and for ensuring accurate postoperative measurement and evaluation.

Preoperative 3D-CT-based Hertel exophthalmometry was performed for Cases 2, 3, 4, and 7, with conventional Hertel exophthalmometry repeated during follow-up (Table 2). Two patients (Cases 5 and 7) underwent follow-up 3D-CT due to improvements in the implant fabricating and design process, specifically the incorporation of barium sulphate (BaSO4) as a radiopacifier into the cement mixing process.

### 2.1. Imaging and Measurement

The minimum imaging requirements include a helical CT with a slice thickness of 0.6 mm or less. Alternatively, a cone-beam CT with a large field of view (FOV) that encompasses both orbits, along with neighbouring cranial and midfacial structures, with a resolution of 100–600 µm voxel size (preferably at a higher resolution), is also acceptable. From these scans, a series of views are generated to assist both the surgeon and the biomedical engineer in planning and implementation [9]. Imaging is therefore essential not only for accurate diagnostics and surgical guidance but also for the digital design process. To this end, imaging data must be exported in raw Digital Imaging and Communications in Medicine (DICOM) format without any reconstruction or reformatting. From this data, the biomedical engineer generates the following views to determine the extent of the orbital floor defect and OZC deformities.

### 2.2. Three-Dimensional Reconstructions

Three-dimensional orbital reconstructions should include a FOV with at least a 5 cm margin extending superiorly above the supraorbital rims to include the glabella, posteriorly to the root of the zygomatic arches and inferiorly to include both maxillae. This FOV enables the generation of multiple views, including a straight frontal view, left and right profile views, and left and right three-quarter views of the traumatised orbit. Three-quarter views are particularly useful for examining the contours of the OZC, identifying subtle variations in shape, and assessing potential abnormalities that might not be as apparent in frontal or lateral views. The ideal angulation for three-quarter views is approximately 30–45 degrees from a straight frontal view.

**Figure 3:**
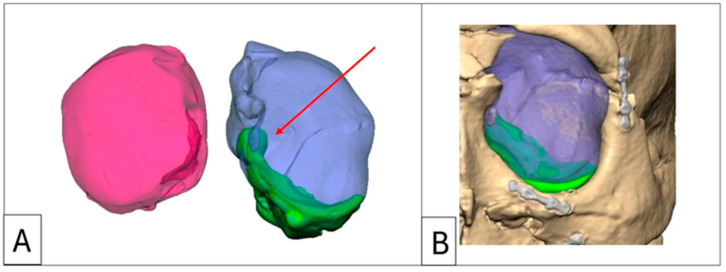
Case 2: Intraorbital volumetric measurements. (**A**) Intraorbital volumetric measurement. Left orbit = 35.98 cm^3^, right orbit = 29.08 cm^3^. (**B**) Increased volume depicted in green, which forms the volume of the enophthalmos wedge. Note the large medial wall component and the re-established posterior medial bulge (red arrow).

Orbitometric measurements in 3D views include intraorbital volumetric assessment using Freeform software, version 2024.0.87, 3D Systems, Rock Hill, SC, USA. This process involves filling the orbits with a virtual material without extending through overt defects in the orbital walls, thereby determining the proposed post-reconstruction orbital volume and geometry. The software then automatically calculates the intraorbital volumes (Figure 3). Based on these values, overcorrection volumes can be calculated and incorporated into the design of full-profile orbital floors or used to select the appropriately sized intraorbital spacer(s). Extraorbital measurements include mirroring, superimposition, and volumetric subtraction (Figure 2).

### 2.3. CT Views

To evaluate the extent of orbital defects, a DICOM viewer in multiplanar view is employed (such software is typically bundled with the scan data or available for download from the radiology department).

**Figure 4:**
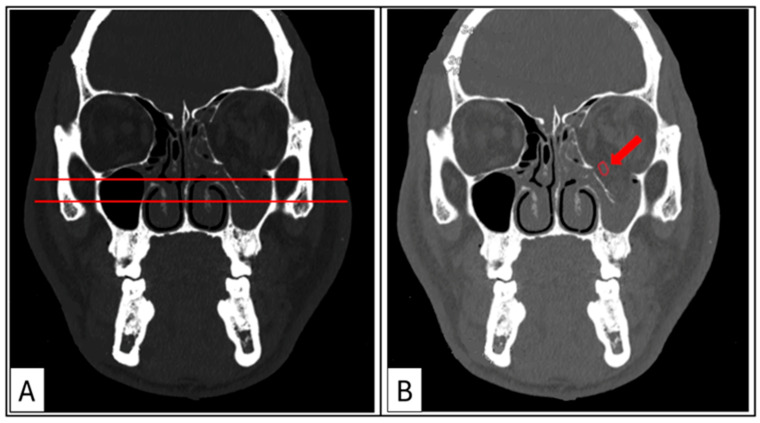
Case 3: Coronal sections. (**A**) Hard-tissue window for the evaluation of the maximal depression point of the orbital floor compared with the contralateral side. (**B**) Soft-tissue window showing rounding of the inferior rectus muscle (red arrow), rupture of the periorbita, and tissue herniation into the maxillary sinus.

Coronal sections (hard- and soft-tissue windows): These provide the most comprehensive visualisation of the orbit, enabling the assessment of the maximal depression point in comparison to the contralateral side and the status of the ocular muscles (Figure 4A,B). The transition zone (TZ) and lateral wall suture (LW) are identified to establish the mid-orbital rim (MR). The maximum width of the orbital floor fracture and/or the maximum height of the medial wall defect is measured and recorded.

**Figure 5:**
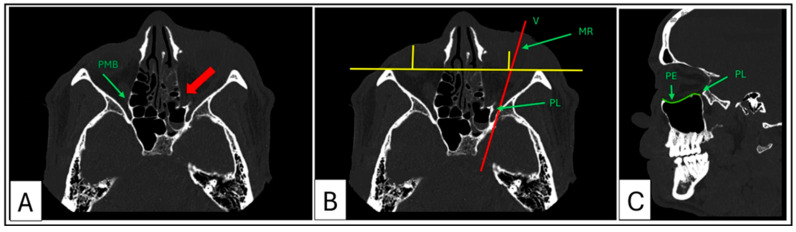
Case 3: Axial sections showing key anatomical landmarks; the posterior medial bulge, posterior ledge, mid-orbital rim, vector, and a paramedian oblique sagittal view. (**A**) The posterior medial bulge on the left side is depressed (red arrow). (**B**) Anatomical landmarks to acquire the paramedian oblique sagittal view vector and yellow lines representing exophthalmometry. (**C**) Key anatomical landmarks in sagittal section; post-entry zone, characteristic lazy S-curve of an intact floor (highlighted in green), and the posterior ledge.

Axial sections: These are used to evaluate the medial orbital wall, particularly the fragile lamina papyracea and the posterior medial bulge (PM) (Figure 5A). The posterior ledge (PL) is identified (located below the optic nerve canal) in this plane, and a vector (V) to the MR is established to acquire the corresponding paramedian oblique sagittal view (Figure 5B,C). Axial views are also used for 3D-CT-based exophthalmometry as described by Park et al. (2019) [10]. This measurement is essential for calculating the proposed volume overcorrection factor required to reposition the globe anteriorly within the orbit and for quantifying postoperative changes in globe position. Additionally, the maximum length of the medial wall fracture is measured and recorded.

Sagittal sections: The paramedian oblique sagittal section is ideal for visualising orbital floor fractures. It is used to identify critical anatomical landmarks such as the post-entry zone (PE), the lazy S-curve, and the PL (Figure 5C). The maximum length of the orbital floor fracture is measured and recorded.

Proposed implant designs are positioned onto these views, forming the foundation of the design report provided by the biomedical engineer. These constitute the minimum views and measurements necessary for accurate planning, design, and surgical implementation.

### 2.4. Bariated Polymethylmethacrylate (PMMA) Bone Cement

Polymethylmethacrylate (PMMA), commonly known as bone cement, has a fascinating history and diverse applications in medicine. PMMA was first introduced in dentistry, where it was used to fabricate a complete denture base in the 1930s [11]. Soon after this, it was used in ophthalmology, primarily as a material for intraocular lenses due to its optical clarity and biocompatibility. Its success in ocular applications quickly highlighted its potential in other surgical specialties [12]. In the 1940s, PMMA found a transformative role in orthopaedics as bone cement. It was utilised to anchor prosthetic implants to bone, particularly in joint replacement surgeries [13]. PMMA’s ability to harden in situ provided mechanical stability, making it an ideal medium for securing implants while simultaneously filling gaps between the bone and prosthetic surfaces. By the 1950s and 1960s, PMMA expanded into neurosurgery, where it became a key material for cranioplasties [14]. Its mouldable nature before polymerisation and quick setting times, combined with its inert properties after setting, allowed for the reconstruction of cranial defects with remarkable durability and precision. In recent years, PMMA has come full circle, returning to the orbit, where it is now employed in reconstructive procedures for traumatic OZC reconstruction. PMMA is valued for its biocompatibility with surrounding tissues and its ability to be shaped intraoperatively using patient-specific, 3D-printed nylon moulds. This historical trajectory underscores the versatility of PMMA as a biomaterial, evolving from its origins in ophthalmology to becoming an indispensable tool in diverse surgical specialties, including this modern resurgence in orbital reconstruction.

**Figure 6:**
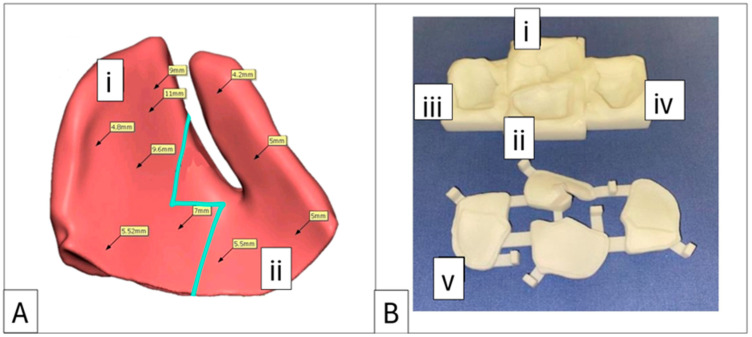
Case 4: Proposed enophthalmos wedge with profile mapping and accompanying 3D-printed nylon mould. (**A**) Implant designed in two sections with a “puzzle piece” joining shape (blue line). Section (i) can be replaced with a 1 mm decrement or increment profile option. (**B**) Negative mould showing sections (i) and (ii) as measured and planned in (**A**), with decrement section (iii) and increment section (iv), which can replace section (i). Label (v) identifies the lid for the base mould.

In all seven cases, the bone cement used for implant fabrication was an industry-standard PMMA bone cement indicated for use in neurosurgery, maxillofacial surgery, and plastic surgery for the repair of cranial and facial bone defects (Synimed, Synicem Cranioplastie, Chamberet, France). This cement carries the CE mark and complies with the relevant European Union health and safety requirements. It may also be substituted with any locally approved PMMA cement. The presentation of the cement is a pouch containing 30 g of powder (polymer) and a glass ampoule with 17 mL of liquid (monomer). Once mixed, it yields 35 cm^3^ of usable cement, far exceeding the requirements of any viable case. This surplus allows for the production of multiple wedges and onlays in small incremental and decremental sizes at no additional cost. These options can account for minor inaccuracies that may accrue during virtual volumetric measurements and potential variances in 3D nylon printing (Figure 6).

Furthermore, since soft-tissue outcomes are more difficult to predict, this option provides precise control in correcting malar projection, offering the surgeon several options for adjustment.

Polymethylmethacrylate cements are radiolucent, a characteristic that offers a distinct advantage in cranioplasty surgery by enabling unobstructed postoperative brain imaging. However, this feature is less favourable in maxillofacial surgery, where confirmation of the implant’s position, either intraoperatively or postoperatively, is preferable. To address this limitation, in Case 7 we incorporated 5.5 g of BaSO_4_ as a radiopacifier into the polymer powder before mixing it with the liquid polymers [15]. Once the polymerisation process was complete, the implant was thoroughly rinsed with sterile saline and implanted.

**Figure 7:**
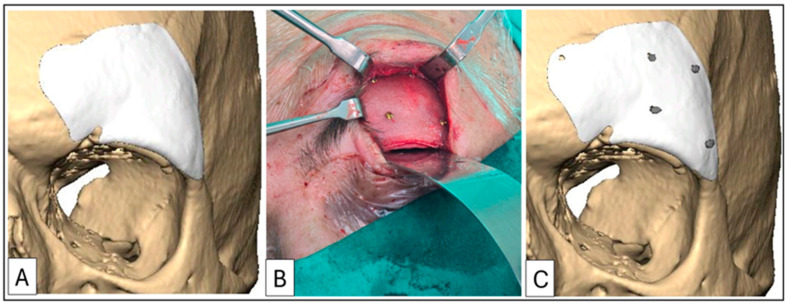
Case 5: A supraorbital rim and frontal bone onlay. (**A**) Planned implant and positioning. (**B**) Intraoperative placement and fixation with four 1.5 mm diameter lag screws (All subsequent PMMA implants were fixated using 1.5 mm diameter screws of a designated length, determined during the planning phase based on preoperative CT measurements). (**C**) Postoperative 3D-CT scan superimposed on planning to confirm correct positioning. Patient 5, a pugilist, required an implant without barium sulphate for enhanced strength. Positioning was confirmed by aligning the lag screws with the planned divots on the implant.

The addition of barium sulphate provided a satisfactory level of radiopacity, allowing for precise confirmation of the implant position via a postoperative 3D-CT scan. Case 6 required an implant without the inclusion of barium sulphate and used planned divots as screw-hole location guides to facilitate postoperative overlaying (Figure 7).

Since onlays are not typically load-bearing, the reduction in fatigue strength caused by the inclusion of barium sulphate is of little consequence, as the biomechanical load on an orbital floor is also very low [15,16]. Furthermore, prior to implantation, the implants may be soaked in an appropriate antibiotic solution, or, alternatively, antibiotics may be incorporated into the mixing procedure. Heat-stable antibiotics such as 5 g of gentamycin, 1–2 g of vancomycin, or 1.0 g of tobramycin can be added during the mixing process [17,18]. This technique is well documented and does not further reduce the implant’s fatigue strength [15].

### 2.5. Nylon 3D-Printed Moulds

A cost-effective and efficient solution for fabricating patient-specific implants is offered by 3D-printed nylon moulds. Nylon, a widely available thermoplastic, is commonly used in 3D printing due to its durability and resistance to heat deformation. This heat resistance is particularly advantageous, as it allows the moulds to withstand the relatively high exothermic temperatures generated during the polymerisation process of PMMA, which can reach 100 °C, as well as the high temperatures involved in standard steam autoclave sterilisation, which typically reaches 134 °C, with a minimum holding time of 3 min.

**Figure 8:**
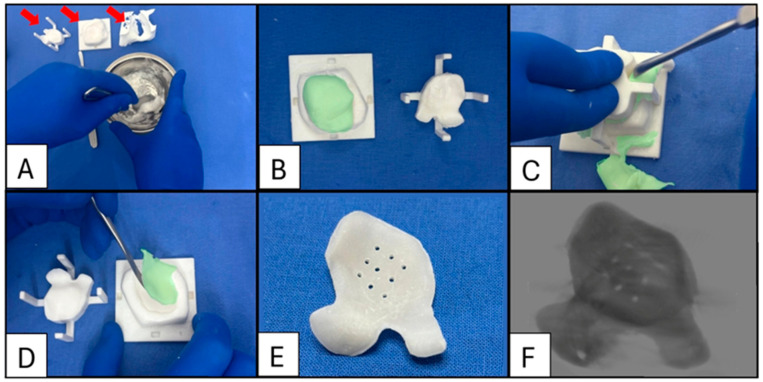
Case 7: Sequence of intraoperative enophthalmos wedge fabrication. (**A**) The polymer is decanted into a bowl and combined with barium sulphate (20% *w*/*w*). Liquid monomers are added and mixed according to the manufacturer’s directions (red arrows indicate the mould lid, mould base, and orbital biomodel to test the implant’s fit). (**B**) The inner surfaces of the base mould and lid are coated with petroleum jelly before populating the mould with approximately 10% excess cement to ensure all air is displaced (The cement is digitally colourised green to aid visualisation). (**C**) The lid is placed and held down with moderate pressure to expel excess cement, which is then removed using an appropriately sized instrument. (**D**) Demoulding: Once the polymerisation process is complete (typically after 20 min), the lid is lifted, and the implant is carefully removed from the base using a sharp-edged instrument. Excess material may be trimmed with a scissor or bur to ensure a smooth edge. (**E**) A series of 1.1 mm diameter drainage holes are drilled over the area corresponding to the orbital floor defect. These holes allow any haematoma that may form to drain into the sinus. (**F**) A 3D-CT scan of the bariated implant.

To facilitate the removal of the cured PMMA implant from the mould, the mould’s interior surfaces are coated with sterile petroleum jelly or lubricant. This coating prevents adhesion between the PMMA and the nylon mould, ensuring easy demoulding and preserving the integrity of both the implant and the mould (Figure 8).

The entire intraoperative implant fabricating process, including the PMMA setting time, takes on average 40 min. The mixing of cement and populating the moulds can be delegated to the surgical assistant or to a suitably trained theatre nurse or scrub technician and therefore does not extend the operating time.

**Figure 9:**
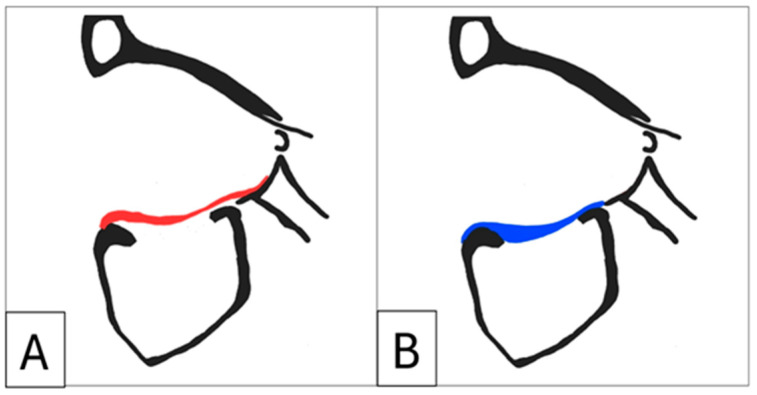
Full-profile orbital floors are designed to eliminate dead space under the implant and to facilitate placement by offering tactile feedback when placed correctly. (**A**) Schematic diagram of a suspension orbital floor compared to a full-profile orbital floor (**B**).

It is recommended that full-profile orbital implants be designed. Some studies describe the use of a “suspension” implant design, which relies on the anterior orbital rim and the posterior ledge for support and creates dead space between the implant and the orbital floor to reduce volume. In contrast, full-profile floor implants achieve stability by resting on existing stable bone and do not necessarily require projections or lips to extend to the posterior ledge. This approach aligns with the standard orthopaedic practice of filling bone voids with antibiotic-loaded PMMA cement (Figure 9) [19]. Moreover, full-profile floors are easier to position, provide tactile feedback when placed correctly, and largely eliminate the need for intraoperative navigation. Additionally, their larger volume allows for implant segmentation, facilitating easier insertion in extensive reconstructions, while also enhancing the overall implant strength.

### 2.6. Design Algorithm

**Figure 10:**
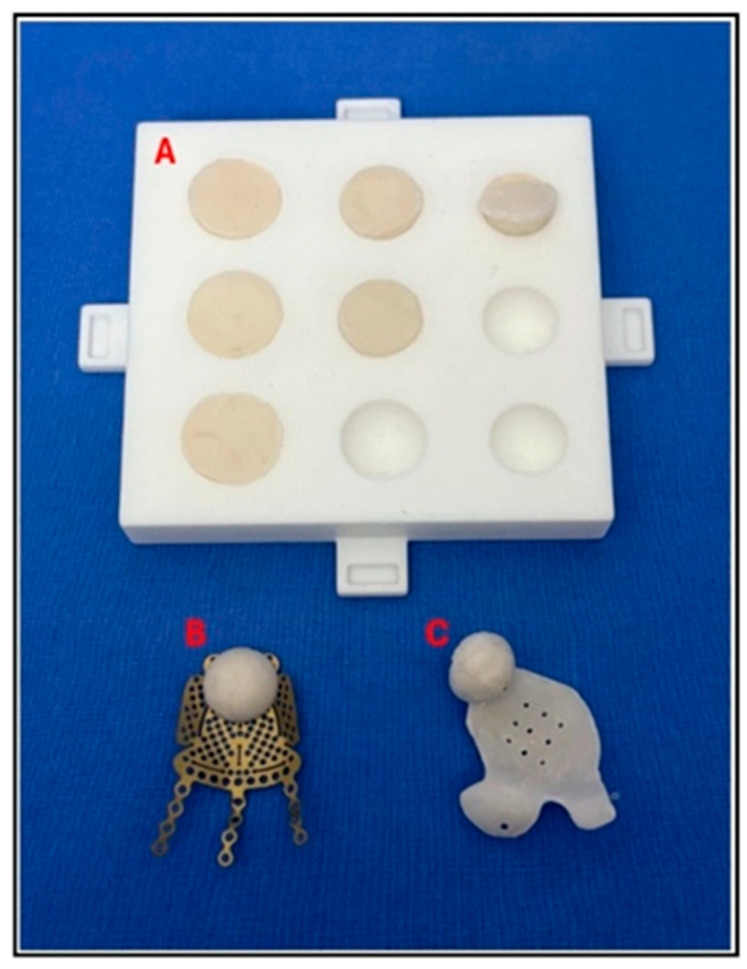
Case 7: (**A**) A mould for making a series of domed intraorbital spacers with volumes of 0.5 cm^3^, 1.0 cm^3^, and 1.5 cm^3^. Spacers can be attached to any designed orbital floor with a 1.5 mm diameter titanium screw. (**B**) A stock titanium orbital floor (Zimmer Biomet, Warsaw, IN, USA) with a 1.0 cm^3^ spacer attached to augment the posterior bulge. (**C**) A patient-matched orbital floor with a 1.0 cm^3^ spacer attached to re-establish a medial posterior bulge.

Implant design requirements for secondary OZC repair are varied and must be addressed without conflating relevant data into a single variable. The primary role of the intraorbital design algorithm is to provide a systematic approach to assessing the design requirements for implants. This includes differentiating between options such as an orbital floor implant (with or without spacers) and an enophthalmos wedge (Figure 10). Additionally, when spacers or wedges are required, the algorithm assists in determining the ideal location(s) and the amount of overcorrection needed.

A clear delineation of implant requirements is established by identifying the primary site(s) of injury, performing meticulous intraorbital volumetric measurements, and considering the time elapsed since injury and primary surgery.

The increase in orbital volume (*Vi*) is determined by subtracting the volume of the uninjured orbit (*Vu*) from the volume of the traumatised orbit (*Vt*) and then calculating the percentage difference:

$$Vi\%=\frac{Vt-Vu}{Vu}*100$$

This value is recorded in the design report and provides the surgeon with critical data to guide clinical decision-making, particularly in determining the extent of the volumetric overcorrection required. Next, volumetric subtraction is performed and profile mapping indicated and shown on the report. If the initial injury involved a fracture of the zygoma resulting in enophthalmos due to an inadequate reduction, the profile mapping should reflect this, with the highest profile measurements located on the lateral wall. Similarly, if the injury site is the medial wall, this would be indicated by increased profile mapping in the same location. Typically, unreduced nasoethmoidal orbital fractures also present with canthal malposition and shortening of the horizontal dimensions of the palpebral fissure [6]. When reconstructing the medial wall, additional volume is incorporated into the implant design to re-establish the posterior medial bulge. Enophthalmos wedges require additional volume to reconstruct the posterior bulge or, if it is deficient, to augment it. In cases of inadequately reduced zygomatic fractures, additional volume is required for the lateral wall.

An increased orbital volume due to large unreduced OZC fractures including an orbital floor fracture requires the use of an enophthalmos wedge to correct the inferior and posterior displacement of the globe. Additional volume is incorporated to restore the level of the orbital floor relative to the contralateral side, and critically, if there is still increased orbital volume after correcting the floor height, then additional volume is added posteriorly in the subperiosteal retrobulbar region, adjacent to the posterior bulge, to reposition the globe anteriorly within the orbit.

In cases of severe trauma to the OZC where more than one orbital wall is involved, computer-aided design should follow a prioritised approach. First, orbital rim defects should be addressed by designing implants and/or onlays that must be incorporated into the anatomy prior to intraorbital volumetric measurement. This step establishes a bony limit for volumetric assessment, and since the rim contributes a “squared” error to the volume of the orbit, it needs to be corrected first; see Grant, Iliff, Manson (1997) [20]. Next, attention should be given to the orbital floor and posterior bulge, followed by the medial wall and posterior medial bulge and finally, the lateral wall.

Studies have demonstrated that 4.2–7.0% of cases involving trauma to the OZC require secondary intervention to address late complications [21,22]. One important variable influencing the incidence of these complications is the time elapsed between the initial injury and primary treatment [21,22,23]. For instance, one study reported that early treatment (within eight days) resulted in no complications, while late treatment (mean delay 10.6 months) was associated with persistent diplopia and enophthalmos rates at 10% and 25%, respectively [22]. There is consensus that early treatment yields optimal outcomes, and this underscores the role of the timing of treatment as a secondary variable that significantly impacts the correction of enophthalmos and diplopia [24,25].

Although Ramieri et al. (2000) concluded that enophthalmos is more closely associated with inadequate volume correction—specifically, the failure to restore the posterior bulge—these findings do not account for late enophthalmos and delayed diplopia [26]. Cohen et al. (2020) demonstrated that orbital fat loss is a contributing factor to late post-traumatic enophthalmos in cases of unrepaired fractures [27]. However, the biological mechanism underlying orbital lipoatrophy secondary to unreduced fractures remains poorly understood. The possibility of a negative pressure forming around the periorbita associated with increased orbital volumes may contribute to changes in adipose tissue, but this is conjecture for now and requires further investigation.

Recent studies indicate that reconstruction to address secondary post-traumatic complications that present as late enophthalmos and delayed diplopia (at a mean of 6 months from time of injury to initial treatment) requires the overcorrection of orbital volume by an average of 8.55% compared to the uninjured contralateral side, with a maximum case increase of 12% [21]. A revised study by Trewhela et al. (2018) further concluded that for every 1 cm^3^ of volume added to a customised orbital implant, the globe is projected 0.7 mm, requiring an average overcorrection of 3.8% [28].

While the variability among the findings reported in the literature necessitates a more detailed analysis in future studies, the present study adopts the following ranges: for late-treatment cases (delayed by 1–3 months), 1.0 mm of projection is achieved for every 1cm^3^ added or an average 7% orbital volume overcorrection. For cases with treatment delayed beyond 6 months, 0.7 mm of projection is expected for every 1 cm^3^ added, or an average overcorrection of 9.55–12% is recommended.

The required overcorrection volume (*OV*) for the implant design is calculated as follows,

$$OV=\left(Vt-Vu\right)+\left(Vu*OF\%\right)$$

The volume of the uninjured orbit (*Vu*) is subtracted from the volume of the traumatised orbit (*Vt*). The resulting difference is then added to the uninjured orbital volume multiplied by the overcorrection factor (*OF*), which may be 7%, 9.55%, or an alternative *OF* determined by the surgeon based on clinical assessment. Finally, the predicted advancement of the globe is determined by multiplying the *OV* by either 0.7 mm or 1 mm, depending on the selected correction parameters. The volumetric subtraction and profile mapping process identifies specific regions requiring overcorrection. It is critical to note that overcorrection should be applied exclusively to the globe-facing surfaces of the implant. Applying the overcorrection volume to all surfaces would result in an implant that fails to conform to the bony contours of the orbit.

### 2.7. Validation and Risks

A three-tier validation protocol was implemented to assess the accuracy, thermal stability, and volumetric fidelity of the patient-specific nylon moulds and resulting PMMA implants. The protocol was designed to align with principles outlined in ISO 14971 (risk management for medical devices) and ISO/ASTM 52901:2017 (additive manufacturing—general principles) [29,30].

The first tier involved printing a series of complex nylon geometries derived from virtual CAD models. These prints were independently measured using calibrated Vernier callipers along the x, y, and z axes and validated against the corresponding virtual digital models. Dimensional accuracy was confirmed, with deviations consistently below 0.1 mm, reflecting a print resolution of 20–30 microns. The second tier tested the resilience of the nylon under sterilisation stress by subjecting the same prints to two full cycles of saturated steam autoclave sterilisation at 134 °C. This simulated a failure–resterilisation event. Post-sterilisation measurements showed no deformation or warping, confirming the thermal stability of the printed shapes. The third tier assessed the fidelity of the complete workflow. Negative moulds were designed and printed from the original virtual test geometries and populated with bariated PMMA cement. After polymerisation, the cement forms were measured manually and scanned using high-resolution 3D-CT. The CT data were converted to standard tessellation language (STL) format and uploaded to an online STL viewer (3dviewer.net), where both dimensional and volumetric conformity to the original virtual digital design were verified. This process confirmed the reliability and reproducibility of the manufacturing workflow from digital planning to intraoperative execution.

The risk assessment of possible contamination of the PMMA cement by the nylon moulds, with possible sequelae, was deemed extremely low for several reasons. First, nylon (validated under ISO 10993) has exceptional biocompatibility due to the amide groups in its chemical structure, which closely resemble those found in natural peptides [31,32,33]. Second, nylon is inherently bio-inert and does not promote any signalling activity within the body’s cells [34]. Its longstanding use in medical applications, most notably in non-resorbable sutures such as ETHICON nylon, commercially available since 1953, further supports its safety profile [35]. Moreover, within the described workflow, moulds are non-implantable and serve only as transient shaping tools with no direct contact with the patient. All moulds undergo validated steam autoclave sterilisation prior to surgery. The interior surfaces of the moulds are coated with sterile petroleum jelly to prevent adhesion but also act as a physical barrier between the PMMA and the nylon substrate. Finally, after demoulding, each implant is manually cleaned and irrigated with sterile saline before implantation.

### 2.8. Regulatory Status of Patient-Specific Implants

The workflow described here was developed under clinical supervision and implemented within a single institution with surgical and manufacturing personnel working in close coordination. However, several important regulatory and legal considerations must be addressed. This study describes the optional off-label admixture of barium sulphate as a radiopacifier, as well as the incorporation of antibiotics into the PMMA cement during mixing. These modifications are performed at the discretion of the surgeon on a case-by-case basis and, most importantly, under individual patient prescription. Although such practices are well supported in the orthopaedic and cranioplasty literature, they are not universally sanctioned and may constitute investigational use under applicable laws [36,37]. This concern may be mitigated by selecting a locally approved PMMA formulation that already includes the desired components, such as PALACOS R + G (Zimmer Biomet, Warsaw, IN, USA) [38].

In the United States, patient-specific implants may qualify under the Custom Device Exemption (CDE), as defined in 21 CFR 812.3(b). Devices falling under this exemption are not subject to FDA premarket approval (such as 510(k) or PMA), provided they are manufactured in response to a written order from a licensed physician, intended for the exclusive use of an individual patient, and produced in quantities not exceeding five units per year of the same design. These devices must carry basic labelling indicating “custom device” status, and while technical documentation must be maintained, it is not submitted to the FDA unless specifically requested. Post-market surveillance (PMS) is encouraged but not formally required under the exemption [39].

In the European Union, patient-specific devices are regulated under EU MDR 2017/745, typically as Custom-Made Devices. Unlike in the U.S., there is no explicit quantity limit, though the devices must not be mass-produced or industrially manufactured. Custom-made devices are exempt from European Conformity (CE) marking, but manufacturers are still required to compile a technical documentation file, which includes the clinical justification, risk analysis, and manufacturing rationale. These devices must be made following a written prescription by a qualified clinician and must include appropriate labelling, such as the name of the prescriber, patient identifier, and a statement that the device is custom-made. Crucially, for Class III custom-made devices (e.g., most implants), involvement of a notified body is mandatory. Additionally, the MDR requires all custom-made devices to be supported by a post-market surveillance system, including a documented PMS plan and vigilance procedures [40]. Together, these frameworks aim to balance clinical flexibility in treating individual patients with regulatory oversight that ensures safety, traceability, and transparency in the manufacturing and use of custom implants.

It is important to note that the responsibility for compliance with regulatory standards applicable to the design and manufacture of patient-specific devices rests with the manufacturing entity. The clinical responsibility lies in prescribing the device appropriately, with legal liability for manufacturing standards residing with the device producer.

In the present study, the 3D-printed nylon moulds were produced by a certified third-party manufacturer (Selective Surgical, Johannesburg, South Africa) with internal regulatory oversight. Institutions replicating this workflow should ensure that the manufacturer, whether a private company or an in-house biomedical engineering department at a university hospital, maintains appropriate regulatory certification, such as ISO 13485 for quality management systems and conformity with local medical device regulations. These bodies are typically responsible for validating the biocompatibility and traceability of the printed devices within the applicable jurisdiction.

## 3. Results

In this retrospective descriptive observational study, the primary predictor variables analysed were enophthalmos, hypoglobus, diplopia, and the patients’ subjective evaluations of cosmesis. Cases 5 and 7 underwent postoperative 3D-CT scans, which were imported into the original planning to confirm correct placement.

Patients presenting with enophthalmos, hypoglobus, and/or diplopia (Cases 2, 3, 4, and 7) experienced a reduction in enophthalmos to 2 mm or less (except Case 7 which was reduced to 3 mm, with hypoglobus reduced to 1 mm), with complete resolution of diplopia in the primary gaze or within the first 20° of the field of vision. The mean follow-up period for all patients was 19.1 months. This extended follow-up suggested that the use of an overcorrection factor in the reconstruction approach was effective in mitigating late-stage enophthalmos. All interviewed patients (Cases 1–7) reported being “satisfied” to “very satisfied” with the aesthetic outcomes using a Likert scale.

No cases of implant displacement, infection, or extrusion were reported within the follow-up period.

## 4. Discussion

The specific challenges presented by each case either confirmed previous findings or contributed to the refinement of the implant design process. Notably, although the literature contains numerous case examples of post-traumatic osteotomies (PTO) for correcting enophthalmos, these procedures are not without shortcomings [41,42]. For instance, Roth et al. (2010) reported that posteriorly displaced zygomaticomaxillary complex fractures cause the orbital floor to buckle posteriorly [43]. When these fractures are reduced to their anatomic position, they may create a significant orbital floor defect [43]. This introduces an unplanned variable, which contradicts the volumetric restoration concept described in this paper. This finding was observed in Case 4, where a PTO was performed.

The primary objective of correcting post-traumatic enophthalmos and OZC deformities is to restore functionality and volume rather than merely replicating the premorbid orbital geometry. Furthermore, in Case 4, a patient-matched PMMA enophthalmos wedge from Selective Surgical, South Africa, and a polyetheretherketone (PEEK) orbitozygomatic onlay from Zimmer Biomet, Warsaw, IN, USA, were used. This introduced logistical challenges in coordinating implant fabrication and mould-printing timelines with the scheduled surgery date. Additionally, the high cost and extended timeframe associated with the PEEK implant’s fabrication and importation into South Africa posed significant constraints. Moreover, the inability to incorporate radiopacifiers or antibiotics into PEEK further limits its suitability for use in complex reconstructions.

For these reasons, in all subsequent cases, implants were designed and fabricated by a single company (Selective Surgical, Johannesburg, South Africa), which completed planning and mould production within one week. Importantly, this streamlined approach allowed for the use of a single implant material (PMMA), improving efficiency and consistency in surgical applications.

Additionally, we confirmed that surgical timing is a crucial variable, with the time elapsed from injury to initial treatment influencing the degree of intraorbital soft tissue changes [22,23].

Practical insights guided refinements to the mould design, including the incorporation of multiple profiles, additional spacers, proposed screw- and drainage-hole divots, segmented implants, and impregnation with barium sulphate and antibiotics.

## 5. Limitations

Several limitations must be acknowledged. These include the small cohort size of this series, the limited sample size of referenced studies, and the evolving nature of the design algorithm. These factors should be considered before implementing the proposed algorithm into standardised practice.

This study explored a novel approach to the intraoperative fabrication of patient-specific, bariated, and antibiotic-loaded PMMA implants. Consequently, fundamental surgical principles—such as complete dissection of the bony orbit, meticulous retrieval of herniated soft tissue, and periodic intraoperative comparison of pupillary dilation with the fellow eye—are acknowledged but not the primary focus of this report. Similarly, the history of OZC surgery, detailed descriptions of surgical approaches, and mandatory examinations—including visual acuity assessment, pupillary response evaluation, and fundoscopy—were not covered, as they fall beyond the scope of this report.

Surgeons and institutions considering similar workflows must ensure compliance with local and national regulatory frameworks. This technique, including modifications to approved materials, may not be legally permissible in all countries. Therefore, replication of the workflow outside of a research or exempted-use context should be preceded by consultation with regulatory authorities, a formal risk assessment, and institutional ethics approval.

## 6. Future Directions

Future refinements to the algorithm should incorporate soft-tissue volumetric measurements, as described by Dinu et al. (2022) [44]. Additionally, preliminary findings from our ongoing research indicate the feasibility of making minor adjustments to the *OF* to account for variables such as injury severity, laterality (in accordance with the axial twist theory), sex, age, and ethnic background [45]. Further studies are required to validate these adjustments and assess their impact on clinical outcomes. Ongoing research aims to refine and expand computational models to enhance implant design algorithms for personalised reconstruction.

## 7. Conclusions

Patient-specific, bariated, and antibiotic-loaded (PSBAL) PMMA enophthalmos wedges and onlays provide an effective and reliable method for restoring orbital volume, correcting enophthalmos and hypoglobus, and improving cosmesis in secondary OZC reconstruction. Additionally, PSBAL-PMMA cement offers several advantages over other biomaterials and autologous grafts, making it suitable for a wide range of OZC reconstructions, from simple patient-matched orbital floors to extensive craniofacial reconstructions.

Moreover, PSBAL-PMMA cement represents an ideal material, addressing many concerns associated with other biomaterials. It offers excellent biocompatibility, exceptional strength, ease of use, zero temperature conduction, either radiopacity or radiolucency, customisable profiles from 0.3 mm, and complex geometries. Given the favourable outcomes observed in this study, patient-matched PMMA implants should be considered a viable option for post-traumatic orbital reconstruction.

## Figures and Tables

**Table 1:** Patient Data: Mechanism of injury, injury sites and side, initial treatment, and revision surgery with patient-specific implant(s).

Patient No	Age	Sex	Mechanism of Injury	Orbitozygomatic Injuries	Side	Initial Surgery	Revision Surgery
1	65	F	Slip and fall (no loss of consciousness)	IR, malar	R	Conservative	Patient-specific IR and malar onlay Lateral tarsal strip canthoplasty
2	26	M	Motor vehicle accident (unrestrained driver)	Nasal, bones Buttress, IR, FZ, Orbital floor, MW	L	Closed reduction ORIF Suprafoil (0.35 mm)	Patient-specific enophthalmos wedge
3	40	M	Interpersonal violence	Orbital floor	L	Suprafoil (0.35 mm)	Patient-specific enophthalmos wedge
4	25	M	Motor vehicle accident (unrestrained passenger)	IR, FZ, zygoma Orbital floor	L	Delayed treatment due to the severity of concomitant injuries	Patient-specific IR, SR, and malar onlay (PEEK) Patient-specific enophthalmos wedge (PMMA)
5	28	M	Sports injury (pugilism)	Supraorbital rim, Frontal bone	L	ORIF	Patient-specific supraorbital rim and frontal bone onlay
6	31	M	Interpersonal violence	FZ, zygoma, IR	L	ORIF	Post-traumatic osteotomy Patient-specific IR onlay
7	32	F	Motor vehicle accident	Frontal bone IR, FZ, Zygoma Orbital floor, MW	L	Decompressive craniectomy ORIF Suprafoil (0.35 mm)	Patient-specific cranioplasty implant Patient-specific enophthalmos wedge and spacers (1 × 1.5 cm^3^ posterior medial bulge and 1 × 1.5 cm^3^ posterior bulge spacers)

M, male; F, female; FZ, frontozygomaticus; IR, infraorbital rim; SR, supraorbital rim; MW, medial wall; ORIF, open reduction internal fixation; L, left; R, right.

**Table 2:** Preoperative investigations and measurements, surgical timing, follow-up period, and postoperative investigations and measurements.

Patient No	Preoperative Examinations	Enophthalmia/Hypoglobus	Interval Between Injury and Initial ORIF	Interval Between Initial ORIF and Secondary Surgery	Follow-Up Period	Postoperative Examinations	Enophthalmos/Hypoglobus
1	3D-CT	--	4 d	3 wk	1 yr 10 mo	Clinical assessment, Likert scale	--
2	HE, 3D-CT	5 mm/3 mm	5 d	4 wk	1 yr	HE, Likert scale	1 mm/0 mm
3	HE, 3D-CT	4 mm/2 mm	5 d	2 wk	4 mo	HE, Likert scale	2 mm/0 mm
4	HE, 3D-CT	5 mm/2 mm	--	4 mo	4 yr 2 mo	HE, Likert scale	2 mm/0 mm
5	3D-CT	--	7 d	3 mo	3 wk	3D-CT, Clinical assessment, Likert scale	--
6	3D-CT	--	5 d	2 wk	2 yr 2 mo	Clinical assessment, Likert scale	--
7	HE, 3D-CT	7 mm/4 mm	2 wk	>6 mo	1 yr 7 mo	HE, 3D-CT, Likert scale	3 mm/1 mm

ORIF, open reduction internal fixation; HE, Hertel exophthalmometry; d, days; wk, weeks; mo, months; yr, year.

## Data Availability

The data presented in this study are available on request from the corresponding author due to privacy concerns.

## Abbreviations

The following abbreviations are used in this manuscript:

3D	Three-dimensional
PMMA	Polymethylmethacrylate
OZC	Orbitozygomatic complex
3D-CT	Three-dimensional computed tomography
OF	Overcorrection factor
BaSO4	Barium sulphate
FOV	Field of view
DICOM	Digital imaging and communications in medicine
TZ	Transition zone
LW	Lateral wall
MR	Mid-orbital rim
PM	Posterior medial bulge
PL	Posterior ledge
V	Vector
PE	Post-entry zone
ISO	International Organization for Standardization
CE	European Conformity
Vi	Increase in orbital volume
Vu	Volume of the uninjured orbit
Vt	Volume of the traumatised orbit
OF	Overcorrection Factor
OV	Overcorrection Volume
CAD	Computer aided design
STL	Standard tessellation language
PTO	Post traumatic osteotomy
PEEK	Polyetheretherketone
PSBAL	Patient-specific bariated and antibiotic-loaded
